# Nikolaus Ballenberger: Evidenzbasierte Assessments in der Muskuloskelettalen Physiotherapie

**DOI:** 10.3205/zma001851

**Published:** 2026-06-15

**Authors:** Kristina Flägel

**Affiliations:** 1 Oldenburg in Holstein, Germany

## Bibliographical details

Nikoilaus Ballenburger

**Evidenzbasierte Assessments in der Muskuloskelettalen Physiotherapie**

Publisher: Elsevier Verlag GmbH, München

Year of publication: 2025, 488 pages, price: € 44.00

ISBN: 978-3-437-46003-6

## Review

The book “Evidence-Based Assessments in Musculoskeletal Physiotherapy” was published in its first edition by Elsevier in 2025. In his foreword, the editor, Prof. Dr. Nikolaus Ballenberger, emphasizes the “(professional) ethical and legal obligation and responsibility to ensure the best possible care to which the patient is entitled”. Fulfilling this requires the use of evidence-based assessments in daily clinical practice – among other reasons – to make treatment progress visible. Consequently, the book lists assessments that are underpinned by studies of sufficient quality.

The book’s structure is divided into a first section describing the theoretical background, authored by Prof. Ballenberger, and a second section covering available assessments for various body regions, as well as clinical prediction rules (CPRs) and patient-reported outcome measures (PROMs). The authors of these chapters are all current students or graduates of the Bachelor’s and Master’s programs at Osnabrück University of Applied Sciences.

The introduction within the first section outlines the significance of assessments for evidence-based physiotherapy. The subsequent chapters establish the groundwork necessary to evaluate the essential characteristics of evidence-based assessments. Specifically, they present the distinguishing features of assessments; types of errors – including their relationship to quality criteria; reliability and validity – including statistical methods for the various forms of reliability and validity; and the generalizability of study results. The sixth chapter of the first section is dedicated to explaining the methodology used to formulate recommendations for the clinical application of the assessments presented in the book’s second section. These assessments were identified through a two-stage systematic literature search. The first step involved identifying assessments for a specific body region for which scientific publications existed; the second step focused on specifically extracting studies pertaining to the quality criteria of these identified assessments. Assessments were included if they were applied to clarify specific musculoskeletal complaints in adults and “potentially allow for inferences regarding pathologically altered and/or painful structures and/or body-region-specific dysfunctions” (p.64). These include not only standalone assessments but also test batteries, PROMs, and CPRs. Assessments based on studies with a high risk of bias – as well as those deemed impractical to apply (i.e., associated with unacceptable time and cost requirements for physiotherapy practice) – are not included in the second part of this book. The clinical utility of the assessments is evaluated based on thresholds for positive and negative likelihood ratios (LR+/-), Pearson and Spearman correlation coefficients, Cronbach’s alpha, intraclass correlation coefficients (ICC), Kappa, Cohen’s effect size, and the area under the curve (AUC). The strength of the recommendation for each assessment takes into account not only the strength of the statistical parameters but also the risk of bias in the underlying studies. A strong recommendation for clinical use requires at least two studies with a low risk of bias that demonstrate clinical utility.

The chapters covering the assessments (cervical spine; jaw and head region; shoulder region; elbow; hand; lumbar spine; sacroiliac joint; hip joint; knee joint; foot and ankle; clinical prediction rules; and patient-reported outcomes) each begin with a brief introduction and a chapter overview. This overview outlines the dimensions addressed within the chapter (e.g., posture, range of motion, and cervical instability in the “cervical spine” chapter), as well as the current evidence base, recommendations, specific considerations, and practical feasibility of the assessments presented on the subsequent pages. For each assessment, the procedure for administration on a patient is described; evidence regarding its validity and reliability is presented in detail using color-coded tables; and recommendations for its clinical application are provided in tabular form, supplemented by a textual summary where appropriate. Finally, each chapter concludes with a tabular summary of the recommendations for all included assessments, accompanied by supplementary textual notes where necessary. Frequently, assessments not explicitly listed are also presented here, and the reasons for their exclusion are discussed. All assessments can be found in the comprehensive index.

Overall, the book is clearly structured and developed with scientific rigor, characterized by a consistent approach to methodology and the presentation of results. With its theoretical background, it offers comprehensive support for navigating the academic landscape of assessments. This is ensured, among other things, through explanations of the terminology used within the English-speaking sphere. The depth of the theoretical background presented is certainly open to discussion, though Prof. Ballenberger notes that this book is not intended to serve as a substitute for specialized textbooks on assessment development or statistical methods. Crucially, the theoretical background ensures that the subsequent chapters on assessments are entirely comprehensible. The illustrative presentation style – for instance, through the use of original studies to exemplify various concepts such as statistical methods for criterion validity – is particularly helpful. Meticulous engagement with the literature, along with appropriate cross-references, enables interested readers to delve deeper into the subject matter should they find the coverage in the foundational section insufficient. Furthermore, the inclusion of quality appraisal tools (e.g., the “quality appraisal of diagnostic reliability studies”) within the first section underscores the book’s rigorous academic standards.

With a consistent emphasis on the necessity of evidence – as well as on strengthening the professional identity and professionalization of the physiotherapy profession – this book is dedicated to the patient and to ensuring their optimal care. It comprehensively fulfills the concept of evidence-based care, which comprises external evidence, internal evidence, and patient preferences: “[…] when assessing the clinical course, patient preference must be strictly observed; that is, depending on the clinical problem, individual priorities must be taken into account. While for Mr. Müller, pain symptoms may be the primary concern, for Ms. Maier, improving quality of life might take precedence” (p.43).

Not least, this book highlights the degree of humility that should guide us as healthcare professionals in our treatment of patients, particularly when we see – here, in black and white – that the available research evidence is often quite limited. Prof. Ballenberger describes this aptly: “[…] the selection of the supposedly best assessments is never a guarantee that consistently correct clinical judgments and decisions will be made. As a therapist, one must always remain aware that every clinical judgment and every decision is potentially fallible” (p.67).

“Evidence-based assessments in musculoskeletal physiotherapy” is not a book one simply reads cover-to-cover out of general thematic interest; rather, it serves as a practical companion for addressing specific clinical questions. It is an invaluable resource for the practicing therapist or physician striving to continuously improve their patient care; for the researcher engaged in the (further) development of assessments or planning the use of evidence-based assessments in clinical studies; and for the student or trainee in the health professions seeking to focus on achieving the most accurate assessment possible of their patients' musculoskeletal systems.

## Competing interests

The author declares that she has no competing interests.

